# Update of Faecal Markers of Inflammation in Children with Cystic Fibrosis

**DOI:** 10.1155/2012/948367

**Published:** 2012-08-21

**Authors:** Jung M. Lee, Steven T. Leach, Tamarah Katz, Andrew S. Day, Adam Jaffe, Chee Y. Ooi

**Affiliations:** ^1^School of Women's and Children's Health, Faculty of Medicine, University of New South Wales, High Street, Sydney, NSW, Australia; ^2^Department of Respiratory Medicine, Sydney Children's Hospital Randwick, Sydney, NSW, Australia; ^3^Department of Paediatrics, University of Otago (Christchurch), Christchurch, New Zealand; ^4^Department of Gastroenterology, Sydney Children's Hospital Randwick, Sydney, NSW, Australia

## Abstract

There is evidence of intestinal inflammation in patients with CF. Intestinal inflammation may negatively impact the nutritional status of patient with CF, which adversely affects pulmonary function and survival. This paper provides an up-to-date review of intestinal inflammation in CF and an evaluation of utility of two specific faecal inflammatory markers (S100A12 and calprotectin).

## 1. Introduction

Cystic fibrosis (CF) is the most common, life-shortening, recessive disease in Caucasians with an average life expectancy of 40 years [1]. In the majority of cases, mortality in CF is due to respiratory failure. CF is caused by mutations in the gene that encodes for the cystic fibrosis transmembrane conductance regulator (CFTR) protein [2, 3]. The CFTR protein functions on the apical surface of epithelial cells as a cyclic AMP-dependent chloride and a bicarbonate channel [4, 5]. Absent or defective CFTR leads to viscous luminal secretions in affected organs particularly in the lungs, intestines, and pancreas. 

The nutritional status of CF patients is a major determinant of pulmonary and survival outcomes. Longitudinal cohort studies in CF report a distinct survival advantage among patients with better nutritional status [6–8]. More specifically, poor nutritional status is not only strongly linked to poorer lung function but was also an independent risk factor for early death in children with CF. Several factors contribute to impaired nutritional status in CF. These include malabsorption, recurrent sinopulmonary infections, increased energy expenditure, and suboptimal intake [9]. The malabsorptive state in CF is likely multifactorial. The primary cause of malabsorption is due to maldigestion from pancreatic exocrine insufficiency. However, children with CF can continue to have malabsorption despite pancreatic enzyme replacement therapy (PERT) administration. It has been previously suggested that the presence of an acidic intestinal milieu, that impairs enzyme activity, contributes to the failure of PERT in nutrient assimilation in CF. More recently, intestinal inflammation has been hypothesized as another contributing factor. 

## 2. Intestinal Inflammation in Cystic Fibrosis

**Figure 1:**
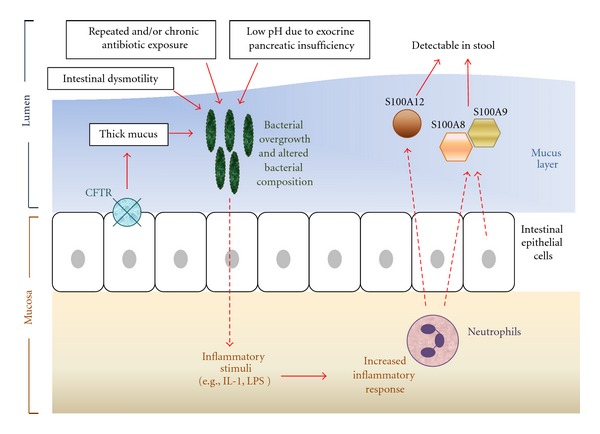
Proposed pathogenesis of intestinal inflammation in cystic fibrosis.

There is increasing evidence of intestinal inflammation in CF. Although the exact underlying mechanism is unknown, there have been several pathogenic mechanisms proposed (Figure 1). As a primary consequence of a defective CFTR, intestinal mucus secretions are viscid and inspissated due to dehydration, are in an acidic milieu, and have altered glycosylation of mucins [10, 11]. Such environmental changes may predispose to alterations in the balance and/or composition of the gut flora (dysbiosis), resulting in mucosal inflammation. Indeed, evidence to support the presence of alterations in gut microbiota (or dysbiosis) has been reported in murine models of CF. Quantitative PCR of bacterial 16S RNA in CF mice revealed a >40-fold increase in bacterial load in the small intestine alone [11]. In CF mice with proven dysbiosis, expression of inflammation-related genes decreased when these mice were treated with antibiotics, hence supporting the link between intestinal dysbiosis and inflammation in CF. Furthermore reduction of intestinal bacteria significantly improved the growth of CF mice but had no effect on growth of wild-type mice [10]. Intestinal dysmotility was also observed in CF mice, and attributed to the further development of dysbiosis and bacterial overgrowth [11]. Secondary factors such as intestinal resection (e.g., due to meconium ileus) or early and/or repeated antibiotic exposure to treat sinopulmonary infection(s) may also influence gut microbial ecology. It has also been suggested that inflammation is induced by an increased and abnormal antigenic load, which is a result of the inadequate digestive enzymes present in the small intestines [12]. 

Smyth et al. [13] previously reported measurements of intestinal inflammatory proteins from whole gut lavage of 21 children with pancreatic insufficient (PI) CF and 12 controls. This study demonstrated increased production of inflammatory biomarkers including albumin, IgG, IgM, interleukin- (IL-) 1*β*, IL-8, neutrophil, elastase, and eosinophilic cationic protein in children with CF. In a separate study that compared duodenal mucosal specimens (obtained endoscopically) from 14 PI CF patients, 20 healthy controls, and 4 non-CF patients with PI chronic pancreatitis, an increased mononuclear cell infiltrate which expressed intercellular adhesion molecule (ICAM)-1, IL-2 receptor alpha (CD25), IL-2, and interferon-*γ* was observed in the lamina propria of duodenal mucosal specimens from CF children [14]. Interestingly, 2 CF patients who had commenced PERT prior to the endoscopy had evidence of intestinal inflammation. Furthermore, small intestinal inflammation was not observed in subjects with non-CF PI chronic pancreatitis, suggesting that PI itself is unlikely the cause of intestinal inflammation. 

Bruzzese and colleagues [15] were the first investigators to evaluate noninvasive methods for measuring intestinal inflammation using faecal calprotectin and rectal nitric oxide production. Mean (SD) faecal calprotectin levels were significantly higher in 30 children with PI CF compared to 30 healthy controls (219 (94) *μ*g/g versus 46 (31) *μ*g/g), but significantly lower than 15 children with active inflammatory bowel disease (IBD) (309 (86) *μ*g/g). However the incidence of intestinal inflammation was similar with 29 of the 30 CF and all 15 of the IBD children having abnormal levels of calprotectin. Twenty of these children with CF, 20 controls, and 15 children with active IBD went on to have rectal nitric oxide production measured by the rectal dialysis bag technique. Similarly, nitric oxide levels were significantly higher in children with CF than controls but did not differ to those with IBD. Bruzzese and colleagues then went on to give 10 CF children a probiotic, *Lactobacillus casei strain GG* (LGG) once a day for 4 weeks and remeasured faecal calprotectin and rectal nitric oxide levels. Notably, both biomarkers of calprotectin and nitric oxide were significantly reduced in 8 and 5 of the 10 children, respectively, suggesting an association between intestinal inflammation and modifications in intestinal microflora. 

In a more recent study, Werlin et al. [16] conducted wireless capsule endoscopy in 41 CF patients including 13 who were pancreatic sufficient (PS), thereby permitting direct inspection of the small intestinal mucosa for endoscopic abnormalities. Signs of small intestinal inflammation include edema, erythema, mucosal breaks, and frank ulceration in 26 of the CF patients (of which 6 were PS). Those with PI generally had more pronounced mucosal changes than those with PS. When faecal calprotectin was obtained from a subset of these CF patients, it was normal in all 9 PS patients (23.2 ug/g) and elevated in those with PI (257.7 ug/g). 

Bruzzese et al. evaluated whether abnormal intestinal microflora may result in an abnormal immune/inflammatory response elsewhere, such as in the lungs [17]. Using a randomized, placebo-controlled, crossover study they looked at whether or not the administration of LGG is associated with a reduction in pulmonary exacerbations in CF. Thirty-eight children and adults with PI CF were randomized to 6 months of LGG supplementation or placebo and then after a 4-week, wash-out period crossed over to the placebo and LGG group respectively. The incidence of pulmonary exacerbations and the number of hospital admissions were significantly higher in the placebo group than the LGG group. Outcomes were consistently better when looking at groups in parallel or longitudinally. A similar study was conducted by Weiss et al. [18]. This study involved 10 patients with PI CF who received probiotics (*Lactobacillus acidophilus, Lactobacillus bulgaricus, Bifidobacterium bifidum, and Streptococcus thermophiles*) every day for 6 months. A significant reduction in pulmonary exacerbation was observed during the 6 months intervention period. Although Bruzzese et al. [17] and Weiss et al. [18] showed a reduction in pulmonary exacerbations, concurrent measurement of biomarkers of intestinal inflammation was not performed. 

In addition, a possible trend between intestinal inflammation (as measured by faecal calprotectin) and nutritional status was reported by Werlin et al. [16]. The interpretations arising from this study, however, were limited by the reporting of mean BMI values despite having a mixed paediatric and adult population. Based on the studies conducted so far, there is convincing evidence that intestinal inflammation is present in CF, particularly those with PI. However the potential clinical implication of this has not been fully explored. Gastrointestinal inflammation is a modifiable factor, which may impair nutrient absorption and, therefore, negatively impact on growth. Not all patients with intestinal inflammation had overt gastrointestinal symptoms and endoscopic evaluation is invasive, costly, and time consuming. The ability to identify and monitor intestinal inflammation noninvasively is therefore advantageous.

## 3. Faecal**  **Inflammatory Markers

As discussed, several biomarkers have been previously used to detect intestinal inflammation in patients with CF. These biomarkers include S100A12, calprotectin (S100A8/S100A9), IL-8, albumin, lactoferrin, IL-1, IgM, IgG, neutrophil elastase, tumour necrosis factor-*α*, and eosinophil cationic protein (ECP) [13, 15, 16]. The S100 proteins, calprotectin and S100A12, will be discussed in detail. 

Both of these S100 biomarkers have been validated for use in another chronic inflammatory condition of the intestines, namely, IBD. Furthermore, both calprotectin and S100A12 have been used as markers of airway and/or systemic inflammation in CF. On the other hand, the other biomarkers listed have been poorly studied in both CF and intestinal inflammation.

### 3.1. S100 Proteins

The human calcium-binding S100 proteins have been proposed to be sensitive markers of inflammation. S100 proteins comprise a family of more than 20 calcium-binding proteins with various intracellular and extracellular roles including calcium sensing, regulation of cell growth, cell migration, and protein phosphorylation [19–22]. A number of S100 proteins, in particular S100A8 (MRP8, calgranulin A), S100A9 (MRP14, calgranulin B) and S100A12 (calgranulin C) have innate immune functions, are expressed in phagocytes [21] and are associated with inflammation. 

### 3.2. Calprotectin (S100A8/S100A9)

The proteins S100A8 and S100A9 are found in granulocytes, monocytes, and early differentiation stages of macrophages and each has individual intracellular and extracellular functions [22]. Furthermore, S100A9 associates with S100A8 in a complex referred to as calprotectin, which is a heterogeneous arrangement of S100A8 and S100A9. Importantly, calprotectin is also induced under inflammatory conditions. Thus, calprotectin is not a disease-specific marker for CF. Nevertheless, over two decades ago the protein complex, calprotectin, was identified in the serum of individuals with CF and was termed the CF-associated antigen (CFA). This complex is a heterodimer that is secreted by a novel pathway and is present systemically or in particular fluids in a variety of inflammatory conditions [23]. S100A8 and S100A9 are found in various cell types, including neutrophils, monocytes, macrophages, dendritic cells, epithelial cells, microvascular endothelial cells, and fibroblasts, generally as a result of activation [24]. The S100A8/S100A9 complex comprises 60% of the cytosolic protein in neutrophils. Inflammatory triggers such as cytokines (e.g., IL-1) or bacterial products (e.g. LPS) activate monocytes and intestinal epithelial cells [25]. This stimulates specific expression and secretion of calprotectin by intestinal epithelial cells resulting in the elevated fecal calprotectin levels in faeces. Calprotectin also activates macrophages with an interaction with toll-like receptor 4 (TLR4) inducing a positive feedback. Calprotectin activates endothelial cells inducing expression of endothelial adhesion molecules. This activation stimulates leucocyte adherence promoting recruitment of leucocytes to inflamed intestinal tissue. TLR4 is activated on neutrophils amplifying inflammation by releasing cytokines (IL-1, TNF-*α*) and reactive oxygen species (ROS). Calprotectin is released by neutrophils from this activation [26]. Furthermore, calprotectin may also alter the gut microbiota by chelating zinc, an essential metabolite for bacteria survival [27].

Studies reporting the use of faecal calprotectin in patients with CF have been already been discussed. Faecal calprotectin has been more extensively evaluated as a biomarker of intestinal inflammation in IBD. Faecal calprotectin is able to discriminate adults with active IBD from those with irritable bowel syndrome with 100% sensitivity and 97% specificity using a cutoff of 30 mg/g [28, 29]. Similar distinctions have also been made for paediatric patients with active IBD [29–31]. von Roon et al. evaluated the diagnostic precision of fecal calprotectin for IBD using prospective studies comparing fecal calprotectin against the histological diagnosis and calculated a sensitivity of 95% and a specificity of 91% for the diagnosis of IBD (versus non-IBD diagnoses) [32]. Furthermore, faecal calprotectin levels were associated with the degree of IBD activity evaluated with clinical, endoscopic, and histological parameters [28–30, 33]. 

In addition, calprotectin has also been measured in the serum and respiratory tract secretions in CF patients. Golden et al.  [34] measured serum calprotectin levels in children with CF, many of who had infective pulmonary exacerbations at the time of measurement. Serum calprotectin levels were higher in the CF group than in healthy controls. It was concluded that serum calprotectin provided a better assessment of acute airway inflammation than traditional serum biomarkers of inflammation (such as white-cell count and C-reactive protein). 

Recent data suggests that there may be interactions between the production of S100 proteins and the underlying genetic abnormalities in CF. Renaud et al. [2] reported potential regulation of the expression of the S100A8 and S100A9 genes by mutations in CFTR. This association supports the possibility that CF may have a primary inflammatory component, due to altered epithelial barrier function and decreased innate immunity at this level. This may occur at the respiratory and gastrointestinal epithelial borders. Thomas et al. [35] also suggested that inflammation is a primary component of CF related to an underlying abnormal immune response. In this study, CF mice (carrying the G551D mutation) had an altered immune response to bacterial products (e.g., LPS). 

### 3.3. S100A12

In contrast to the broad expression pattern of calprotectin, S100A12 is more restricted primarily to granulocytes [21, 22, 24]. S100A12 acts independently without S100A8 or S100A9 during calcium dependent signalling. S100A12 is exclusively released after inflammatory activation of granulocytes. S100A12 exerts inflammatory effects on a variety of cell types through binding to the receptor of advanced glycation end products (RAGE). RAGE ligation activates Nuclear-Factor-(NF-) *κ*B and related intracellular signalling pathway with the consequent production of pro-inflammatory mediators [36]. The interaction with RAGE and receptors on endothelial cells also induces leucocyte adhesion and transmigration. Stimulation of granulocytes in an autocrine fashion results in release of cytokines like IL-1, TNF, and ROS production. This positive feedback triggers an additional release of S100A12 by mucosal and intraluminal granulocytes. S100A12 is therefore closely related to granulocyte activation.

The role of S100A12 in intestinal inflammation in CF has not been fully evaluated [37]. Similar to calprotectin, the role of S100A12 as a biomarker of intestinal inflammation has been better defined in IBD, where faecal S00A12 is shown to be a valid and reliable noninvasive biomarker of intestinal inflammation. S100A12 was elevated in stools collected from children shown to have extensive bowel inflammation (*n* = 20; 142.4 ± 125 mg/kg) compared to controls with no bowel condition (*n* = 24; 2.8 ± 4.8 mg/kg) (*P* < 0.0001) [38]. Furthermore, receiver operator curve analysis indicated that faecal S100A12 distinguished gut inflammation with 96% sensitivity and 92% specificity with cutoff of 10 mg/kg and area under the curve of 99.2%. In addition, S100A12 levels were stable in stool even when stool samples were stored at room temperature for up to 7 days. A further study expanded upon these findings for both S100A12 (and calprotectin) [31]. Children with intestinal symptoms were enrolled prospectively with faecal S100A12 and calprotectin measured. Half of these children were diagnosed with active IBD upon review of mucosal biopsies. Both S100A12 (median 55.2 mg/kg) and calprotectin (1265 mg/kg) were elevated in the children with active IBD (*n* = 31) in contrast to the children without any inflammatory changes present (*n* = 30) (S100A12 median 1.1 mg/kg, *P* < 0.0001; calprotectin median 30.5 mg/kg; *P* < 0.0001). Furthermore, S100A12 (using a cutoff of 10 mg/kg) gave a sensitivity and specificity of 97%, respectively, for the detection of inflammation whereas calprotectin (cutoff 50 mg/kg) gave a sensitivity of 100% and specificity of 67%. 

In CF, Foell et al. [21] ascertained that S100A12 levels were raised in the serum and sputum of affected patients. The population included individuals with end-stage lung disease and others having an infective exacerbation (rather than young children). Mucosal expression of S100A12 was increased in lung biopsy samples (immunohistochemistry) and levels of S100A12 were increased in serum and sputum samples. Furthermore, the levels of this protein were increased at the time of an infective exacerbation and fell after successful treatment, potentially playing the role of a surrogate serum marker for infectious exacerbations.

## 4. Areas for Future Research 

Future work is required to better understand the underlying mechanisms in the development of intestinal inflammation. As alterations in the gut microbiota have been implicated, the use of next generation high throughput sequencing technology to investigate the gut microbiota in CF should be considered. The direct effects of intestinal inflammation on nutritional and pulmonary status have not yet been fully investigated. Further work is also required to determine the most reliable biomarker(s) of intestinal inflammation in CF. In addition, the roles of targeted anti-inflammatory therapies for the gut as well as use of pro- and prebiotics require further evaluation, with promising clinical implications.

## 5. Conclusion

In conclusion, there is clear evidence of intestinal inflammation in CF, particularly those with PI. Intestinal inflammation may negatively impact the nutritional status of patient with CF, which in turn adversely affects pulmonary function and survival. Small therapeutic studies using probiotics have shown reduction in pulmonary infective exacerbations and have lead to reductions in levels of faecal inflammatory markers. Faecal biomarkers of inflammation, especially calprotectin and S100A12, have promising roles in the identification of these patients. Greater insights into the intestinal milieu in CF and its systemic implications are much needed.

## References

[1] Yankaskas JR, Marshall BC, Sufian B, Simon RH, Rodman D (2004). Cystic fibrosis adult care: consensus conference report. *Chest*.

[2] Renaud W, Merten M, Figarella C (1994). Increased coexpression of CFTR and S100 calcium binding proteins MRP8 and MRP14 mRNAs in cystic fibrosis human tracheal gland cells. *Biochemical and Biophysical Research Communications*.

[3] Riordan JR, Rommens JM, Kerem BS (1989). Identification of the cystic fibrosis gene: cloning and characterization of complementary DNA. *Science*.

[4] Quinton PM (1983). Chloride impermeability in cystic fibrosis. *Nature*.

[5] Reddy MM, Quinton PM (2001). Selective activation of cystic fibrosis transmembrane conductance regulator Cl- and HCO3- conductances. *Journal of the Pancreas*.

[6] Corey M, McLaughlin FJ, Williams M, Levison H (1988). A comparison of survival, growth, and pulmonary function in patients with cystic fibrosis in Boston and Toronto. *Journal of Clinical Epidemiology*.

[7] Lai HC, Corey M, Fitzsimmons S, Kosorok MR, Farrell PM (1999). Comparison of growth status of patients with cystic fibrosis between the United States and Canada. *American Journal of Clinical Nutrition*.

[8] Corey M, Gaskin K, Durie P, Levison H, Forstner G (1984). Improved prognosis in CF patients with normal fat absorption. *Journal of Pediatric Gastroenterology and Nutrition*.

[9] Pencharz PB, Durie PR (2000). Pathogenesis of malnutrition in cystic fibrosis, and its treatment. *Clinical Nutrition*.

[10] Norkina O, Burnett TG, De Lisle RC (2004). Bacterial overgrowth in the cystic fibrosis transmembrane conductance regulator null mouse small intestine. *Infection and Immunity*.

[11] De Lisle RC (2007). Altered transit and bacterial overgrowth in the cystic fibrosis mouse small intestine. *American Journal of Physiology*.

[12] Ballinger AB, Camacho-Hübner C, Croft NM (2001). Growth failure and intestinal inflammation. *QJM*.

[13] Smyth RL, Croft NM, O’Hea U, Marshall TG, Ferguson A (2000). Intestinal inflammation in cystic fibrosis. *Archives of Disease in Childhood*.

[14] Raia V, Maiuri L, de Ritis G (2000). Evidence of chronic inflammation in morphologically normal small intestine of cystic fibrosis patients. *Pediatric Research*.

[15] Bruzzese E, Raia V, Gaudiello G (2004). Intestinal inflammation is a frequent feature of cystic fibrosis and is reduced by probiotic administration. *Alimentary Pharmacology and Therapeutics*.

[16] Werlin SL, Benuri-Silbiger I, Kerem E (2010). Evidence of intestinal inflammation in patients with cystic fibrosis. *Journal of Pediatric Gastroenterology and Nutrition*.

[17] Bruzzese E, Raia V, Spagnuolo MI (2007). Effect of Lactobacillus GG supplementation on pulmonary exacerbations in patients with cystic fibrosis: a pilot study. *Clinical Nutrition*.

[18] Weiss B, Bujanover Y, Yahav Y, Vilozni D, Fireman E, Efrati O (2010). Probiotic supplementation affects pulmonary exacerbations in patients with cystic fibrosis: a pilot study. *Pediatric Pulmonology*.

[19] Tibble JA, Sigthorsson G, Bridger S, Fagerhol MK, Bjarnason I (2000). Surrogate markers of intestinal inflammation are predictive of relapse in patients with inflammatory bowel disease. *Gastroenterology*.

[20] Golden BE, Clohessy PA, Russell G, Fagerhol MK (1996). Calprotectin as a marker of inflammation in cystic fibrosis. *Archives of Disease in Childhood*.

[21] Foell D, Seeliger S, Vogl T (2003). Expression of S100A12 (EN-RAGE) in cystic fibrosis. *Thorax*.

[22] Donato R (2003). Intracellular and extracellular roles of S100 proteins. *Microscopy Research and Technique*.

[23] Foell D, Wittkowski H, Vogl T, Roth J (2007). S100 proteins expressed in phagocytes: a novel group of damage-associated molecular pattern molecules. *Journal of Leukocyte Biology*.

[24] M NS (1998). The S100 family of multipurpose calcium-binding proteins. *Journal of Cutaneous Pathology*.

[25] Foell D, Wittkowski H, Roth J (2009). Monitoring disease activity by stool analyses: from occult blood to molecular markers of intestinal inflammation and damage. *Gut*.

[26] Brandtzaeg P, Gabrielsen TO, Dale I, Muller F, Steinbakk M, Fagerhol MK (1995). The leucocyte protein L1 (calprotectin): a putative nonspecific defence factor at epithelial surfaces. *Advances in Experimental Medicine and Biology*.

[27] Sohnle PG, Hunter MJ, Hahn B, Chazin WJ (2000). Zinc-reversible antimicrobial activity of recombinant calprotectin (migration inhibitory factor-related proteins 8 and 14). *Journal of Infectious Diseases*.

[28] Tibble J, Teahon K, Thjodleifsson B (2000). A simple method for assessing intestinal inflammation in Crohn’s disease. *Gut*.

[29] Bunn SK, Bisset WM, Main MJ, Golden BE (2001). Fecal calprotectin as a measure of disease activity in childhood inflammatory bowel disease. *Journal of Pediatric Gastroenterology and Nutrition*.

[30] Bunn SK, Bisset WM, Main MJC, Gray ES, Olson S, Golden BE (2001). Fecal calprotectin: validation as a noninvasive measure of bowel inflammation in childhood inflammatory bowel disease. *Journal of Pediatric Gastroenterology and Nutrition*.

[31] Sidler MA, Leach ST, Day AS (2008). Fecal S100A12 and fecal calprotectin as noninvasive markers for inflammatory bowel disease in children. *Inflammatory Bowel Diseases*.

[32] von Roon AC, Karamountzos L, Purkayastha S (2007). Diagnostic precision of fecal calprotectin for inflammatory bowel disease and colorectal malignancy. *American Journal of Gastroenterology*.

[33] Costa F, Mumolo MG, Bellini M (2003). Role of faecal calprotectin as non-invasive marker of intestinal inflammation. *Digestive and Liver Disease*.

[34] Golden BE, Clohessy PA, Russell G, Fagerhol MK (1996). Calprotectin as a marker of inflammation in cystic fibrosis. *Archives of Disease in Childhood*.

[35] Thomas GR, Costelloe EA, Lunn DP (2000). G551D cystic fibrosis mice exhibit abnormal regulation of inflammation in lungs and macrophages. *Journal of Immunology*.

[36] Hofmann MA, Drury S, Fu C (1999). RAGE mediates a novel proinflammatory axis: a central cell surface receptor for S100/calgranulin polypeptides. *Cell*.

[37] Ooi CY, Lee JM, Leach S, Katz T, Day AS, Jaffe A (2012). Intestinal inflammation in CF: stool markers and correlation with pancreatic enzymes. *Journal of Cystic Fibrosis*.

[38] de Jong NSH, Leach ST, Day AS (2006). Fecal S100A12: a novel noninvasive marker in children with Crohn’s disease. *Inflammatory Bowel Diseases*.

